# ROOM to Grow, a Mobile Well-Being Intervention for University Students: Overview of the Design Process and Outcomes

**DOI:** 10.2196/63325

**Published:** 2025-12-17

**Authors:** Tajda Laure, Camila Villegas Meija, Danielle Remmerswaal, Djameela Dulloo, Ruth Van der Hallen, Birgit Mayer, Marianne Littel, Bram Dierckx, Jeroen S Legerstee, Rutger C M E Engels, Marilisa Boffo

**Affiliations:** 1 Department of Psychology, Education, and Child Studies Erasmus School of Social and Behavioural Sciences Erasmus University Rotterdam Rotterdam The Netherlands; 2 Erasmus University Rotterdam Rotterdam The Netherlands; 3 Department of Child and Adolescent Psychiatry/Psychology Erasmus MC - Sophia Children’s Hospital Rotterdam, South Holland The Netherlands; 4 Academic Center for Child and Adolescent Psychiatry and Specialized Youth Care Levvel Amsterdam, North Holland The Netherlands; 5 University of Amsterdam Research Institute of Child Development and Education Amsterdam The Netherlands

**Keywords:** mobile intervention, mHealth, micro-intervention, mental health, emotion regulation, self-awareness, transdiagnostic, skill transfer, student well-being, university students

## Abstract

**Background:**

University students are facing a multitude of challenges and an increase in mental health issues that affect their academic performance and overall well-being. In response, Erasmus University Rotterdam launched the Student Wellbeing Programme in 2019, offering comprehensive, tailored support through a stepped-care framework to enhance student success and well-being. One of the tools developed for students is ROOM to Grow, an anonymous and accessible preventative mental health app.

**Objective:**

This paper describes the process and outcomes of ROOM’s design and development, guided by the Centre for eHealth Research (CeHRes) road map and privacy-by-design principles, and highlights the lessons learned throughout this process.

**Methods:**

This paper describes the first 4 phases of the CeHRes Road map: contextual inquiry, value specification, design, and operationalization. It outlines the population (ie, stakeholders), methods (ie, literature reviews, expert groups, cognitive walkthroughs, interviews, experimental designs), and outcomes of each phase.

**Results:**

The most common mental health struggles among our target population were stress, anxiety symptoms, perfectionistic tendencies, and loneliness. Students often recognized these issues only once they became overwhelming. Regarding digital tools, students seek credible content specific to their experiences, as well as adaptable and intuitive systems; they are mindful of data privacy and aim to reduce their screen time. ROOM was developed to address the diverse needs and preferences of university students through a transdiagnostic approach to mental health. It targets emotion regulation (ER) skills and self-awareness, which underlie the mental health challenges experienced by our target users. ROOM comprises 26 brief exercises (ie, micro-interventions) that support the development and use of adaptive ER. The exercises incorporate techniques from various therapeutic approaches (ie, self-compassion, positive psychology, mindfulness, cognitive behavioral therapy, and acceptance and commitment therapy) to accommodate students’ diverse content preferences. To help students recognize their struggles earlier, ROOM includes a mood tracker and a self-assessment module with questionnaires that evaluate both traits (eg, perfectionistic tendencies) and states (eg, stress levels), providing personalized feedback. ROOM further implements an intelligent recommender system that connects users to relevant content, enhancing the tool’s personalization and responsiveness to users’ needs. As students aim to minimize screen time, ROOM’s goal is not prolonged app use but the application of ER skills in real life, supported by features that facilitate skill transfer into everyday settings. Finally, ROOM was developed within a “privacy-by-design” framework to address students’ privacy concerns, implementing strict privacy and security regulatory standards.

**Conclusions:**

Compromises were necessary to balance user needs, resource constraints, and privacy-by-design and security standards, often limiting ROOM’s interactivity. Other challenges included simplifying complex psychological concepts into brief formats, fostering interdisciplinary collaboration, balancing academic rigor with industry production pace, and operating with fixed resources while maintaining an iterative process. This paper may serve as a primer for designing transdiagnostic, adaptive mental health interventions for youth, blending therapeutic approaches and promoting skill transfer.

## Introduction

### Background

In the last decade, universities have been compelled to evolve beyond their traditional roles. Historically centered on imparting knowledge and academic skills, universities are now taking on greater responsibilities to support the holistic development of their students [1]. This shift is driven by growing recognition of the multifaceted challenges university students face today. There is greater emphasis on the development of soft and personal skills that are crucial for participation in the modern workforce and integration into today’s society. This expanded educational mission reflects the need to move beyond merely study success—often gauged by grade point average, dropout rates, and study career progression—to student success, which encompasses personal (eg, development of a value system to inform future decisions, self-efficacy) and professional (eg, effective communication, problem-solving) growth in addition to academic achievements [2,3].

The necessity for this shift has partly arisen from increasing reports of mental health problems among university students, coupled with a noticeable shortage of available resources [4,5]. Seminal reports indicate that one-third of university students globally experience mental health problems such as depression, anxiety, and substance abuse, yet only 1 in 5 receive adequate help [4,5]. Mental health problems not only affect the individuals themselves but also have a detrimental impact on society. In particular, they are directly linked to poorer academic performance and higher dropout rates [6,7], negatively influencing individual well-being [8] as well as career and financial prospects [9]. On a broader scale, when students falter, society loses out on individuals equipped with essential skills vital for economic growth and innovation, posing challenges in meeting the demand for highly skilled workers required for sustainable development and maintaining competitiveness in the global market [10].

### What Does It Mean to Be a Student Nowadays?

The current generation of students faces pressures from global concerns such as climate change [11], COVID-19, housing crises [12], and geopolitical tensions, alongside academic pressures [13], adding layers of complexity to their university experience and overall development. Most university students fall within a developmental phase known as emerging adulthood [14], during which individuals transition from adolescence to full adulthood. Emerging adulthood typically spans from the late teens to the mid- to late twenties and is characterized by identity exploration, instability, self-focus, a sense of in-betweenness, and a feeling of possibilities [14,15]. This period often involves frequent relocations and significant changes in social and personal identities. For university students, the typical milestones of this life stage—such as moving away from family and gaining greater independence—are further compounded by the added pressures of academic demands and the potential accumulation of student debt [16]. Although the transition to university presents an opportunity for positive personal development, the inherent instability of this period, combined with academic and other pressures, may create conditions that contribute to the development of mental health issues. A shift toward a more holistic approach to student success within academia aligns well with the priorities of Generation Z (1997-2012), who comprise the majority of today’s university students. Generation Z tends to be less focused on traditional career paths and more attuned to the broader implications of their roles in society [3]. Considering the present-day context, universities need to expand their focus beyond academic success and career progression to also foster self-exploration and personal growth among students [3]. By doing so, they can cultivate well-rounded individuals who are better prepared to navigate both the workforce and life’s challenges.

### Erasmus University Rotterdam: Student Wellbeing Programme

In 2019, Erasmus University Rotterdam (EUR), located in Rotterdam, The Netherlands, launched the Student Wellbeing Programme [17], a university-wide initiative designed to foster student success by supporting their well-being and personal development, grounded in a whole-system perspective. This multifaceted, research-driven programme considers contextual, organizational, and individual factors that are crucial for student success. It systematically addresses student well-being through tailored support services that account for the diverse needs and characteristics of its student body, while also facilitating systemic changes to the university’s role in students’ lives. The programme integrates services, events, initiatives, and policies within the university’s educational and professional services infrastructure (ie, a so-called chain of care). Such a comprehensive, grassroots approach can better reach students and provide them with effective, holistic support [18]. Central to this initiative is a stepped-care framework [19,20]—a structured system of support services and interventions tailored to the severity and nature of students’ needs. For example, the programme offers universal prevention and self-help measures, skill-building workshops, mental health awareness events, and spaces designed to facilitate social connection. One of the interventions at this level is *ROOM,* a mobile app designed to provide tools that foster mental health and self-growth, empowering students to manage their well-being proactively. For those requiring more intensive support, the stepped-care model extends to increasingly targeted and specialized services, such as academic and well-being coaching and access to university psychologist services.

### The Case for Building Yet Another Mobile App

eHealth solutions provide accessible, scalable, anonymous, and adaptable support, making them a promising avenue for enhancing the delivery of mental health services in both preventive and therapeutic settings [21]. Literature suggests that online interventions can effectively improve emotional health and reduce symptoms of anxiety and depression among university students [22-25]. While today’s students are more open to discussing mental health compared with previous generations, many hesitate to seek help due to stigma, time and financial limitations, and a preference for self-managing their mental health challenges [24,26]. At the same time, this generation engages with digital tools such as mobile phones more than any other [27,28]. Providing students with a mobile mental health tool (ie, ROOM) represents a viable approach to addressing some of the barriers to seeking psychological help (as suggested by Andersson and Titov [29]; Ebert et al [30]). Moreover, a tool implemented within the university ecosystem serves as an accessible, low-threshold entry point to the stepped-care model established by the EUR Student Wellbeing Programme.

Given the increasing popularity of digital health solutions, the current landscape of mobile mental health offers a wide range of off-the-shelf solutions for users. These apps hold considerable promise, as they can be cost-effective and provide timely support and privacy to their users [31]. Despite their popularity, however, these tools vary in quality and frequently exhibit several limitations [31,32]. For instance, while some apps incorporate evidence-based therapeutic techniques such as cognitive behavioral therapy (CBT) or mindfulness practices, only a very small fraction have been empirically tested and validated [33]. Of the approximately 15,000 commercial mental health apps available on the market, only 3%-4% are evidence based [34]. Research on mental health apps in the university context further indicates that only a few commercially available apps meet the specific needs of university students [35-37]. University students prefer digital tools that are easy to use, integrate credible and high-quality educational content, allow customization (such as notification settings), offer personalization (ie, responsiveness to their needs), are cost-free or include free trial periods, and implement robust security measures to protect their data privacy [35,36,38]. However, these features are often lacking in currently available apps. More rigorous research and development are needed to create effective online interventions for this target population, with an emphasis on user experience (UX) and engagement to ensure sustainable implementation within universities—an aspect often overlooked [23-25].

Recognizing (1) the potential of digital tools and the inherent familiarity of university students with mobile devices, (2) students’ behavioral patterns and preferences regarding help-seeking and mental health management, and (3) the current state of existing digital mental health solutions, we sought to leverage mobile technology to develop a service that is accessible, private, evidence based, and scientifically validated. This service is aligned with students’ needs and designed to integrate within the broader university support ecosystem, helping students cultivate the skills necessary to better manage their mental health.

### Our Approach to Mobile Intervention Development

To develop this mobile intervention, we formed a multidisciplinary team that brought together diverse knowledge bases and areas of expertise. The team included clinical and health psychologists, behavioral researchers, visual designers, game and interaction designers, behavior change intervention designers, human-computer interaction specialists, information technology developers, research methodologists, machine learning engineers, and data scientists. Master’s students enrolled in clinical, health, and positive psychology programs also joined the team to conduct research and assist in developing intervention materials.

In designing our mobile intervention, we adhered to the Centre for eHealth Research (CeHRes) road map, a comprehensive framework for developing eHealth solutions [39,40]. This approach is grounded in human-centered design principles, persuasive systems design, business modeling, and empirical evidence [39,41]. It departs from traditional linear approaches to designing digital interventions and products (eg, waterfall development methods) and instead proposes a progressive, cyclical design process involving iterative assessment and design cycles across 5 development phases, aligning more closely with Agile development methods: (1) contextual inquiry (ie, assessing the problem, context, barriers and opportunities, and stakeholder roles), (2) value specification (ie, establishing guidelines to align the development process with stakeholder needs), (3) design (ie, developing and refining prototypes through iterative testing to ensure relevance, acceptability, and usability among stakeholders), (4) operationalization (ie, finalizing and implementing the eHealth technology), and (5) summative evaluation (ie, evaluating the impact and uptake of the eHealth technology; see Multimedia Appendix 1 for more details [40]). Within this framework, users and other stakeholders are actively involved as co-designers of the end solution, which evolves from a conceptual prototype to an increasingly functional and complex system. Decisions are research driven, evaluated, and cyclically improved until the product is finalized.

### Objectives

The goal of this paper is 2-fold: to present the developed solution and to share our journey and lessons learned from creating a mobile mental health app for university students. We aim to provide intervention developers working with this target population with actionable insights to create relevant and engaging mental health tools, as well as to serve as a primer on developing adaptive digital interventions.

**Figure 1:**
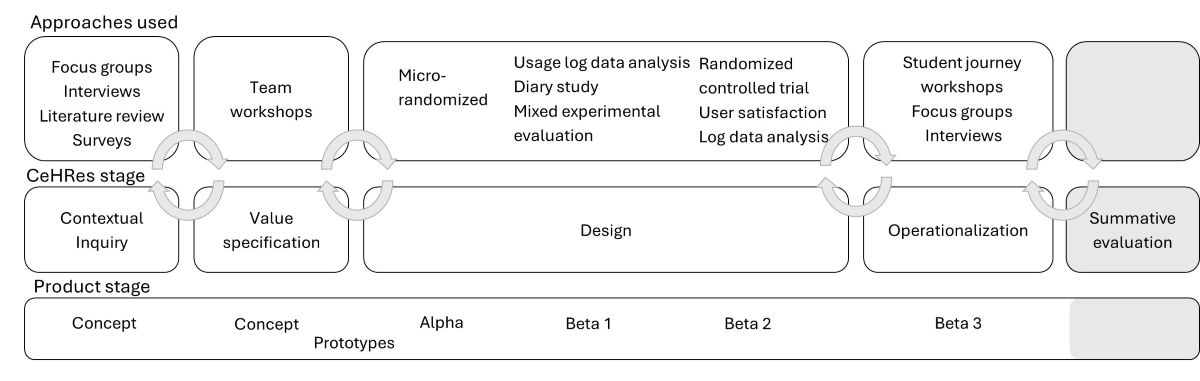
Centre for eHealth Research (CeHRes) road map approach to ROOM’s (the intervention) development.

The focus of this paper is on the first 4 phases of the CeHRes road map: contextual inquiry, value specification, design, and operationalization (as depicted in Figure 1), excluding the summative evaluation phase, which is reported elsewhere [42]. Although the intervention development process was iterative, we present the information linearly, aligned with the road map phases, for clarity. In the following sections, we outline the methods and main outcomes for the first 3 phases. The paper concludes with a description of the developed product, ROOM, and its integration within the EUR ecosystem, including an overview of our implementation strategy (ie, the operationalization phase). Finally, the discussion synthesizes the main insights and lessons learned from the intervention development process.

## Contextual Inquiry

### Methods

The goal of this phase was to develop an understanding of university students, their context, and their challenges; to identify other relevant stakeholders; and to determine their needs to ensure seamless integration of the final product within the broader system in which the tool will be used. In this phase, we addressed the following research questions:

- What personal or academic challenges do students at EUR experience? How and where do they seek support, and what could be improved in terms of available support at the university?
- What is the prevalence of mental health problems among EUR students?
- How do students relate to technology and well-being, and how do they spend their time?
- What aspects of digital media and technology do students value most?
- Which mental health approaches would be most helpful for students in managing their mental health during their time at university?

Participants in the contextual inquiry phase included EUR students (aged 18-30); staff members who were in direct contact with students and aware of their struggles (eg, university psychologists, study advisors, student counselors); and researchers and mental health professionals working with youth and experienced in mental health approaches.

To address the research questions, we used a mixed-methods approach that combined qualitative and quantitative techniques, including focus groups, semistructured interviews, large-scale surveys, and comprehensive literature and desk research. Data analysis methods were tailored to each methodology and research question, employing techniques such as descriptive statistics, affinity mapping, and summary reporting. Key outcomes were identified through triangulation of insights across all research activities.

### Results

#### Overview

A total of 3827 students participated in the contextual inquiry phase (female: n=2564, 67%; male: n=1231, 32.17%; nonbinary or preferred not to disclose: n=21, 0.55%; and gender not disclosed: n=11, 0.29%), along with 17 university staff in student-facing roles and 8 experts in mental health and technology (see Table 1). Further research details and results from the contextual inquiry phase are available in the Open Science Framework (OSF) environment [43].

**Table 1:** Overview of research goals, evaluation methods, participant information, and analysis.

Research question and method		Participant group	Number	Key details	Analysis	
**Struggles and support**						
	7 focus groups	Students	33	21 (64%) female and 12 (36%) male	Descriptive statistics and the participants’ input were summarized and documented in subsequent reports.	
	3 focus groups	University staff	17	13 study advisors, 3 student psychologists, and 1 student counselor	Descriptive statistics	
**Mental and physical healthnology, well-being, activities**						
	Large-scale survey [44]	Students	3769	2536 female (67.29%), 1212 male (32.16%), and 21 nonbinary (0.56%)	Descriptive statistics	
**Technology, well-being, and activities**						
	Semistructured interviews	Students	11	No demographic details provided	Affinity mapping	
	Semistructured interviews	Students	9	4 (44%) female, 5 (56%) male	Affinity mapping	
	A focus group	Students	5	3 (60%) female, 2 (40%) male	The participants’ input was summarized and documented in subsequent reports.	
**Therapeutic approaches**						
	2 focus groups	Experts	8	1 psychiatrist, 1 health care psychologist, 2 clinical psychology researchers/mindfulness practitioners, 1 clinical psychology researcher/therapist, and 2 mental health researchers	The participants’ input was summarized and documented in subsequent reports.	

#### Outcomes of the Contextual Inquiry

##### Psychological and Situational Challenges Among University Students

Results from the focus groups with students (n=33) and university staff in student-facing roles (n=17), as well as from the large-scale survey [44] (n=3769), indicated that students face significant internal and external challenges. Psychologically, they commonly struggle with anxiety, depression, stress, loneliness, and the pressure to perform and achieve perfection, often compounded by inadequate study skills. Typically, students recognize these issues only once they become overwhelming, suggesting limited mental health literacy and self-awareness. Many also report poor service accessibility, such as long waiting times, insufficient information, and confusion about available resources. At the same time, situational difficulties—such as financial constraints and high academic expectations—further contribute to their stress.

##### University Students’ Perspectives on Technology, Digital Media, and Well-Being

Research exploring how students relate to technology and well-being, their leisure activities, and what they value in digital media (including interviews with 20 students and a focus group with 5) yielded 3 key insights: students’ experimental preferences, engagement dilemmas, and implementation considerations.

###### Key Experiential Preferences

Students expressed distinct preferences regarding digital tools. First, they emphasized the need for credible, trustworthy content that resonates with their personal experiences. Second, they placed strong importance on privacy and data security, expecting digital tools to be intuitive, adaptable to their needs, require minimal user input, and use relatable, nonpatronizing language. They expressed a clear dislike for social comparisons (ie, comparing their progress or achievements with those of their peers) and valued their time, preferring activities that yield clear, quick, and tangible results. Finally, this group often seeks to reduce screen time due to screen-related stress, demonstrating a preference for minimizing online interactions [45].

###### The Engagement Dilemma

We found that users were saturated with online content and actively attempted to limit their phone usage [43]. However, digital behavior change interventions (DBCIs) require sufficient user engagement to be effective [46]. This highlighted conflicting requirements that needed to be addressed: *How can a DBCI engage users without relying on sustained app usage?* Engagement was therefore redefined beyond mere app interaction, focusing instead on the active application of skills acquired from the app in real-world contexts, guided by the concept of *learning transfer* [42,43].

###### Implementation Considerations

Findings from the Contextual Inquiry phase revealed that students needs and receptiveness to well-being support or technology vary across individuals, phases of the academic year, and years of study. Key periods—such as the start of the academic year, examination periods, and seasonal changes—shape these fluctuations, with notable differences observed between students in different years of study and between national and international students. While some students proactively manage their stress, others are more reactive, seeking support primarily during moments of crisis. Students prefer light, low-effort interactions on mobile devices, reserving longer tasks (eg, journaling) for computers, and are most likely to engage with phones in the mornings, evenings, and during commutes. They often hesitate to seek formal support, typically turning first to friends or family. Additionally, some students hold negative perceptions of the university, viewing it as a culture of overwork with limited care. These varying attitudes and levels of receptiveness may directly influence the app’s acceptability and uptake, highlighting the importance of accounting for these factors when designing the app’s content and communication strategy.

#### Identifying a Suitable Mental Health Approach to Support University Students: Transdiagnostic Approach

First, a pool of potential intervention topics was created, including stress management, emotion regulation (ER), social skills, goal setting, and self-awareness. Next, by triangulating data gathered from the literature review and expert focus groups (n=8), candidates for therapeutic approaches were identified. These included positive psychology [47], mindfulness [48], acceptance and commitment therapy (ACT) [49], self-compassion [50], and CBT [51]. The intervention approaches were tiered from less to more intensive based on problem severity—starting with *positive psychology* for simple mood improvements, progressing to *self-compassion and ACT* for mild to moderate mental health problems, and escalating to *CBT* for more severe issues (see Villegas et al [43]). Initially, the idea was to target specific problems such as anxiety and depressive symptoms using distinct therapeutic approaches. However, to accommodate such a heterogeneous target population, a more parsimonious approach was needed to make the app more flexible and approachable. Therefore, we diverged from traditional problem-specific methods and adopted a transdiagnostic approach, targeting common factors underlying the various mental health challenges experienced by university students. Emotion (dys)regulation was identified as a suitable target, as it emerges as a key factor across a wide spectrum of mental health problems [52]. ER is a complex process involving the initiation, inhibition, or modification of one’s emotional state or behavior in specific contexts [53]. It encompasses various strategies, including choosing or altering the situations one engages in, redirecting attention, reappraising situations, and adjusting one’s emotional or behavioral responses accordingly. Fostering ER skills can lead to improved well-being, interpersonal relationships, and resilience [18].

## Value Specification

### Methods

During the value specification phase, our objective was to identify aspects of the tool that would add value for end users and translate these into specific system requirements. Using the value specification approach outlined in the CeHRes road map [54], we identified recurring themes from the contextual inquiry results and extracted items that would provide value to key stakeholders (ie, students). These were then converted into actionable requirements covering the following: *content* (ie, what the intervention should provide); *UX* (ie, interaction with the content, intervention look and feel); *technical aspects* (ie, app functionalities and back-end system); and *implementation and organizational aspects* (ie, integration of the tool within the university ecosystem and compliance with operational, legal, privacy, and other regulatory frameworks) [54]. These requirements were prioritized based on their relevance to users and other stakeholders (eg, university regulations), as well as financial and technical limitations. A final list of requirements was then created to guide subsequent development. The identified values and requirements were continuously refined in the following phase as we designed, tested, and learned more about our users and available resources.

### Results

The value specification process resulted in themes that were translated into 36 values, which were then operationalized into 175 requirements. A full overview of these requirements is available in the OSF [43]. To maintain conciseness, Table 2 presents an excerpt of the most important themes, subthemes, and related values and requirements that informed ROOM’s creation and its implementation within the broader university ecosystem.

Based on the value and requirements specification, the tool was designed to focus on 2 key areas: improving *self-awareness*—to help students recognize when and why they are struggling (and, when necessary, prompt them to seek support)—and *enhancing ER.* The emphasis was on concise content and a design that is responsive to users’ needs while offering the freedom to engage through different modalities. In terms of implementation, the focus was on maintaining strong security and privacy standards, creating a tool that is visually and functionally distinct from EUR branding and educational platforms to build student trust and separate academic matters from personal mental health, and developing a tailored communication strategy that leverages student ambassadors and aligns with the student journey to ensure accessibility throughout the academic year.

**Table 2:** Summary of key themes, subthemes, and values from the contextual inquiry, mapped to content, UX^a^, and technical and organizational requirements informing intervention design.

Themes	Subtheme	Value to the user and stakeholders	Requirements
Common mental health struggles among EUR^b^ students	Anxiety/rumination/worry, comparison pressure, perfectionism, and fear of failure	Getting techniques to deal with negative thoughts and feelings that might be maladaptive	Content: The app contains exercises that train adaptive emotional regulation skills.
Lack of self-awareness	Students do not know when to get help or what is normal stress, anxiety, and rumination	Having the space and tools to be more self-aware	Content: The app allows students to engage in assessments to understand themselves better, including their traits, states, and psychological mechanisms.
Time sensitivity	Getting something in return for invested time, fast, “time is precious,” reducing phone time.	Learning in easy, quick spurts of time	UX: Interactions and exercises should be short or be segmented into smaller chunks.
Personalized content	Like reduced and curated content, dislike generic nonapplicable content.	Getting content that feels relevant and personal.	UX: The app provides tailored recommendations of activities relevant to the user’s intentions.
Tone and content preference	Undesirable—“cringe” or over-the-top content; dislike condescending tones; like well-researched, evidence-based information; need for validation and compassion; sensitive to pressure/judgment; empathetic vibes; calming music/audio guides when stressed.	Down-to-earth yet science-based advice to deal with emotions. Feeling cared for and not pressured.	UX: Content is candid and to the point. The voice is not rigid or too silly. Content: Scientific sources are included in the app. Content and UX: Content acknowledges societal and academic pressures and provides feedback that feels compassionate and validating. Content: Content emphasizes the humanness of the user (and avoids overtly quantifying the user).
Relationship with screen time	Dislike the addictiveness of social media; dislike notifications; technostress—tired/saturated.	Having the option to follow exercises without needing to access the app.	Content and UX: The app contains content and interactions that support users in practicing, applying, and remembering skills offscreen [42,43].
Visual preferences	Aesthetics are important; “Good Vibes”	Learning from a tool that is aesthetically pleasing and calming.	UX: The aesthetic is “laid back”—resembles chill-hop and other study/relax YouTube channels familiar to the users.
Privacy considerations	Mistrust technology that feels “creepy” or “knows too much”; international and university standards for privacy and security.	A system that prioritizes privacy by design.	Technical: User log data are stored for research or system monitoring purposes only and is done so anonymously. Full usage and users’ data are stored locally on the user’s phone (ie, use of a privacy-by-design development approach). Organizational: The system must adhere to and pass review for relevant privacy and security standards.
Peer endorsement versus university skepticism	Students value and trust advice from other students; the university is seen as a source of stress and is distrusted	Introducing the tool by someone students trust Separation of the well-being tool from the university branding Separation of mental health and personal development from academic performance and studying	Organizational: Student ambassadors promote the tool UX: The tool has a distinct visual style that is separate from the university branding Organizational: The tool is disjointed from the university’s branding and educational support environments.
Attitudes to well-being	Interest in personal development and preventive approaches to well-being; attention to well-being only in reaction to distress	Being aware of the tool’s availability and being able to easily access it when the student’s interest/need arises The communication about the tool is tailored to the receptivity of different student groups Universal and indicated prevention	Content and organizational: The tool and related marketing strategy integrate positive mental health framing and avoid use of language implying pathology (eg, instead of “distorted thoughts,” the term “unhelpful thoughts” is used). Content: The tool provides resources for seeking specialized help when necessary, directing students to the support available through the university’s stepped care model. Organizational: The tool is introduced to students as they are starting university. Organizational: Students are referred to the tool while waiting for an appointment with university psychologists.

^a^UX: user experience.

^b^EUR: Erasmus University Rotterdam.

## Design

### Methods

**Figure 2:**
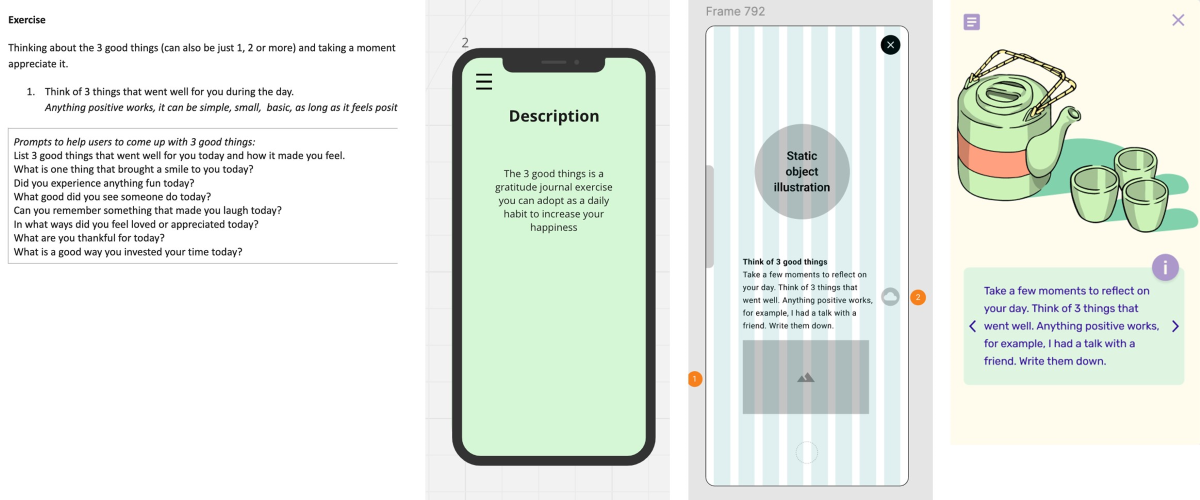
Excerpts from ROOM’s versions from concept to final product: the raw content, an early prototype of the content, an early prototype of interactions, and the latest version (beta I).

Following the value specification requirements, we developed, evaluated, and iterated multiple versions of ROOM at different fidelity levels. The development process comprised 4 main stages: (1) early content and concept prototypes; (2) high-fidelity clickable prototypes; and 2 fully functional app versions—(3) alpha and (4) beta (ie, fully coded mobile apps; see Figure 2).

In the initial stages of prototype development, the focus was primarily on content. During this phase, formative evaluations were conducted by mental health experts to assess the appropriateness, adequacy, and accuracy of the materials. Concurrently, university students reviewed the content for clarity and acceptability. Observational pilot studies explored whether engagement with the intervention content produced changes in targeted skills or moods, aiming to assess improvements in cognitive defusion, cognitive restructuring, self-compassion, mindfulness, and emotional states.

Clickable prototypes were used to assess the acceptability and appeal of the app’s concept and content among students. These evaluations also examined the app’s visual and interaction design to ensure alignment with user expectations and to enhance the overall UX.

The alpha version was developed to experimentally evaluate the effects of the exercises on target outcomes—specifically, users’ emotional states immediately after completing an exercise (proximal effects)—and to explore more distal effects on distress and ER skills over time [55,56]. The beta version was a more comprehensive iteration of the app, incorporating all key components of the final architecture (ie, exercises, self-assessment module, recommender system). It was designed to test features supporting the application of learned techniques in users’ daily lives (ie, learning transfer elements) [43].

Evaluations were conducted with varying levels of rigor—ranging from rapid iteration tests to longer experimental studies. The methods employed at this stage included both qualitative (ie, semistructured interviews, focus groups, design sessions, diary studies) and quantitative approaches (ie, micro-randomized trials and randomized controlled trial designs [A/B testing]).

### Results

#### Overview

In the design phase, a total of 692 EUR students participated, of whom 506 (73.1%) were female, 168 (24.3%) male, and 18 (2.6%) nonbinary or preferred not to disclose. This phase also involved 30 experts, including psychiatrists, psychologists, researcher-practitioners, and digital health specialists. An overview of the research is provided in Table 3. Detailed research methods and results from the design phase are available in the OSF environment [43].

**Table 3:** Overview of key research phases in the design stage: goals, methods, participant information, and analysis.

Fidelity stage, research goal, and method			Participants	Data analysis
**Early concept (low-fidelity)**				
	**Expert evaluation of intervention content**			
		4 expert focus groups	1 psychiatrist, 1 health care psychologist, 3 researcher-practitioners, and 2 digital mental health researchers	Input was summarized and documented in subsequent reports
		Semistructured interviews	4 psychologists	Thematic analysis
	**Evaluating the efficacy of content on key targets**			
		6 quasi-experimental pre-post studies (mixed methods)	192 EUR^a^ students: 139 (72.4%) female, 48 (25.0%) male, and 5 (2.6%) nonbinary	Paired *t* test or Wilcoxon signed rank test
	**Qualitative evaluation of content acceptability and clarity**			
		3 think-aloud sessions and 6 semistructured interviews	85 EUR students: 55 (65%) female, 26 (31%) male, and 4 (5%) nonbinary	Thematic analysis
		Informal user experience interviews	Informal check-in (sample not detailed)	Input was summarized and documented in subsequent reports
**General user engagement**				
	**Explore onscreen and offscreen user engagement**			
		Semistructured interviews	30 in total: 10 mental health professionals, 9 digital experts, and 11 DBCI^b^ users	Grounded theory analysis
**High-fidelity prototype**				
	Review acceptability of content, design, and interactions	Think-aloud sessions + user experience interviews (clickable prototype)	15 EUR students: 9 (60%) female, 4 (27%) male, and 2 (13%) nonbinary	Input was summarized and documented in subsequent reports
	Assess likelihood of usage and skill transfer [43]	5 co-design workshops (high-fidelity prototype)	10 EUR students: 9 (90%) female and 1 (10%) male	Input was summarized and documented in subsequent reports
**Alpha**				
	Evaluate effects on emotional states (micro-randomized trial)	Micro-randomized trial + semistructured interviews	146 EUR students: 119 (81.5%) female, 24 (16.4%) male, and 3 (2.1%) nonbinary; a subsample of 18 in interviews: 11 female and 7 male	Weighted and centered least-squares analysis, paired *t* test, and Wilcoxon signed rank test (see Laure et al [56])
**Beta I**				
	Test effectiveness and acceptability of transfer elements [43]	A/B testing on ROOM versions	231 EUR students: 168 (72.7%) female, 60 (26.0%) male, and 3 (1.3%) no disclosure	Mixed-effects linear models and Wilcoxon signed rank test
	Evaluate experience with learning transfer [43]	Diary study + follow-up interviews	13 EUR students: 7 (54%) female, 5 (38%) male, and 1 (8%) nonbinary	Thematic analysis
	Evaluate ROOM app usage patterns	Usage log data analysis (A/B + diary participants)	62 A/B study participants + 13 diary study participants	Log data analysis

^a^EUR: Erasmus University Rotterdam.

^b^DBCI: digital behavior change intervention.

#### Summary of Key Outcomes of the Formative Evaluations

##### Low-Fidelity Prototype Phase (Early Design and Concept Testing)

Expert focus groups and interviews (n=11) indicated that the intervention content was accurate, appropriate for the targeted problems, and sufficiently faithful to the original therapeutic approaches. Experts recommended incorporating compassionate debriefing messages, including disclaimers that the app does not replace professional therapy, tailoring examples to university life, addressing privacy concerns, and using relatable, unpolished examples to avoid reinforcing perfectionism. They also emphasized the importance of expanding the research to include more male participants, as most early users were female.

Quasi-experimental pre-post studies (n=192) using low-fidelity prototypes (delivered via Qualtrics, Miro boards, or slides) indicated that the exercises improved proximal outcomes, including cognitive defusion, thought believability and discomfort, emotional states, and self-compassion. However, effects on trait-level characteristics were less consistent: one study reported improvements in cognitive defusion after 5 days, whereas another did not, and mindfulness levels remained unchanged [43]. Qualitative research with students (n=85) indicated that participants understood and were able to carry out the presented techniques. Students appreciated the short length of the exercises and the psychoeducational explanations, which helped make abstract concepts more concrete. They also emphasized the need for repetition and longer-term practice to sustain benefits, rather than relying solely on brief interventions. Challenges included emotional distress triggered by certain exercises—particularly those targeting unhelpful thinking patterns—highlighting the need for postexercise debriefs, occasional confusion over complex metaphors, and feelings of being overwhelmed by the volume of text.

##### High-Fidelity Clickable Prototype Phase (Interactive App Design and User Testing)

Evaluation of the high-fidelity clickable prototype using think-aloud methods and interviews (n=15), which simulated the app’s visual design, navigation, animations, and interactions, indicated that users found the app’s aesthetics appealing and the therapeutic content engaging. However, they experienced difficulties with nonintuitive navigation, unclear icons, hidden background information, and audio or animation timing that occasionally made interactions uncomfortable. Users frequently overlooked key elements, such as the “info” button, which disrupted the intended flow of exercises. These findings highlighted the need for clearer navigation, more intuitive interaction design, and enhanced visual cues to guide users smoothly through the exercises.

Co-design workshops (n=10) provided detailed feedback on features related to learning transfer [43]. Action-planning tools (eg, if-then plans) and body-based cues (eg, hand gestures) were perceived as practical and directly applicable to real-life situations [43]. By contrast, social-sharing features (eg, inviting friends to join exercises) were generally rejected, as participants preferred to engage with the app individually and privately. Users favored short, playful exercises that could be easily integrated into daily routines, supported by habit-formation tools such as reminders or personalized challenges, while avoiding complex or cognitively demanding tasks. They further emphasized that more complex or emotionally challenging techniques required repeated, guided practice rather than immediate off-screen transfer [43].

##### Alpha Prototype Phase (Structured Testing With Micro-Randomized Trial)

The alpha version, which included 20 ER exercises, was tested in a micro-randomized trial (n=146) over 21 days. Participants completed exercises twice daily, in the morning and evening, and were subsequently interviewed about their UX (n=18) [55,56]. The trial demonstrated high adherence to intervention completion, with an average completion rate of 65.98% (4,046/6,132), likely supported by monetary incentives for completing daily assessments. Results indicated immediate positive effects on emotional states following the exercises, along with gradual improvements in ER and stress symptoms over the intervention period; depressive and anxiety symptoms remained stable. Qualitative interviews revealed that, while some participants appreciated the structured delivery and felt motivated by the imposed schedule, others found it restrictive and preferred greater autonomy. Exercises lasting longer than 7 minutes were most frequently left incomplete, and those requiring participants to speak aloud or change their environment received mixed feedback. Breathing exercises were the most liked, whereas cognitive defusion exercises were the least liked. Qualitative findings also identified notifications and context-sensitive recommendations as among the most commonly expressed user needs [55,56].

##### Beta Prototype Phase (A/B Testing and Transfer Effectiveness)

The beta I version expanded the app to include 24 exercises and learning transfer elements [43], along with a self-assessment module and a recommendation system based on data from the micro-randomized trial (alpha prototype). A/B testing (n=231) compared app versions with and without transfer elements, focusing on their effects on ER skills and the application of learned skills off-screen [43]. Overall, app usage was low, with an average of only 2.5 completed exercises and brief durations of active engagement. However, the data suggested that the version incorporating transfer elements facilitated earlier application of skills in real-life contexts [43].

A diary study (n=13) evaluating transfer elements [43] showed that participants applied skills learned in the app using transfer elements, such as action plans and environmental cues (eg, stickers) [43]. Participants often simplified the techniques, favoring short, easy practices with immediate effects—such as breathing or grounding exercises—over more complex ones. Perceived positive changes included learning new coping strategies, increased self-compassion, and momentary boosts in positive affect. Barriers to transferring skills to real-life contexts included cognitive demands, forgetting action plans or the purpose of environmental cues (eg, stickers), and feeling overwhelmed by lengthy or complex techniques [43].

User log data indicated that active users (n=75, who participated in the A/B and diary studies) completed an average of 6 exercises and saved the objects earned by completing exercises in the virtual room. Exercises were generally rated positively, with an average likeability score of 73.7/100 and perceived helpfulness of 68.6/100. While 66 users accessed the self-assessment module, only 39 completed a survey. Perfectionism was the most frequently selected topic. Longer questionnaires were often abandoned. Users reported that the app required frequent log-ins, which they found frustrating, and that the overall structure was not user-friendly. Specifically, many struggled to explore content beyond what was highlighted by the recommender system. Additionally, the app lacked features to track progress or revisit past exercises and action plans. The app’s key feature of facilitating the application of learned strategies outside the app (ie, learning transfer) [43] was not presented in a way that felt actionable or salient to users.

#### Outcome of the Design Phase: ROOM to Grow

##### Overview of the ROOM App and Its Core Features

Based on the results of the design phase, we refined the app to create a functional early version, referred to as a minimum viable product. This section summarizes the key features of the ROOM app, including intervention targets, content, engagement strategies, and UX. Detailed descriptions of the components, rationale, underlying theories, and the user journey are available in the OSF environment [43].

##### Intervention Content and Targets

ROOM is a mobile-based, transdiagnostic mental health intervention targeting ER through short (3-10 minutes) behavior change exercises grounded in mindfulness, self-compassion, positive psychology, ACT, and CBT. Each approach addresses different stages of the ER process; for example, cognitive restructuring helps users reframe triggering situations, while self-compassion supports the modulation of emotional responses. The variety of approaches caters to the diverse needs and preferences of university students, allowing them to explore and identify strategies that work best for them. Beyond ER, ROOM is designed to enhance self-awareness. Recognizing that students often delay addressing mental health issues, the app includes a brief monitoring feature that evaluates 7 emotional states (see OSF [43]) and longer questionnaires assessing both transient psychological states (eg, depressive symptoms) and trait-level characteristics (eg, perfectionism). The app identifies risk and protective factors and educates users on topics such as perfectionism, depression, and self-efficacy, while providing personalized feedback.

##### Engagement Strategy

First, students perceived mobile phone use as a source of stress and sought to minimize screen time. Second, expert feedback emphasized the importance of designing content that supports the application of learned skills beyond the app [43]. Third, although users desired autonomy, our studies showed that complete freedom in app usage tended to reduce engagement. To meet the needs of this target group, ROOM is designed to promote real-life skill application while limiting prolonged screen time [43]. To achieve its intended impact, users are encouraged to engage with the app for at least 21 days—based on findings from the alpha version study showing improvements in ER skills and stress [55]—exploring various content, discovering what works best for them, building well-being habits, and mastering the skills learned in the exercises. In line with this, ROOM includes (1) short, actionable therapeutic content for immediate use; (2) a gamified collectible system enabling quick access to favorite exercises from the home screen; (3) features that support the application of learned techniques in real-world contexts (learning transfer) [42,43]; and (4) a 21-day challenge that prompts users to monitor their mood daily and try 1 exercise per day.

##### User Experience

The goal was to design an intuitive, aesthetically pleasing, user-friendly, and time-efficient interface responsive to users’ needs. We prioritized credible, evidence-based content presented in short, focused segments to accommodate limited attention spans. The visual design drew inspiration from Chill Hop, a style popular with the target audience. To avoid overwhelming users, additional details are nested under “insights” and “info” buttons, allowing users to control the amount of content they access.

Each exercise and questionnaire explains its relevance and includes practical, student-focused examples. An intelligent recommendation system addresses users’ need for responsiveness and personalization, connecting them to content that aligns with their individual needs and preferences. The recommender system learns and adapts based on user input while adhering to a privacy-by-design principle. It uses a federated machine learning model, which trains directly on users’ mobile devices, keeping data private by sharing only anonymized model updates with a cloud-based model aggregator (see Laure et al [42] and Villegas Mejia et al [43] for more details). To address students’ privacy concerns, no invasive tracking (eg, geo-location) is employed.

Lastly, to ensure user safety and provide a positive end-to-end experience, the app incorporates several safety measures. For example, rules embedded in the recommender system prevent the suggestion of certain exercise types based on reported moods. The app also provides reassurance after completing exercises or questionnaires through debriefing information. Additionally, it includes up-to-date information on relevant resources, such as university psychologists or general practitioners, for users who may require further support.

## Operationalization

The planning for the integration and dissemination of ROOM within the EUR context was intertwined throughout the development process, particularly during the design phase, when highly refined prototypes were tested before reaching the minimum viable product. Below, we describe ROOM’s implementation within the EUR ecosystem and the strategy for reaching the end users (ie, students).

### Technology—Context Implementation

First, the app is integrated into the broader EUR Student Wellbeing Programme’s chain of care, leveraging its visibility and awareness among most EUR students. Second, considerable effort was devoted to building strong relationships with the university’s faculties, student support services, and information technology services, which supported both the research and the development of the tool. As a result, they have been aware of the initiative and committed to supporting ROOM’s implementation at EUR. Third, ROOM was developed in alignment with both internal university and international standards and regulations. In particular, it adheres to the privacy-by-design framework [57], ensuring that data protection is integrated into the app architecture from its inception. Data privacy was also highlighted by students as an important consideration (see Table 3). Compliance with data and technology regulations (ie, European Union General Data Protection Regulation [EU GDPR] [58], EU Artificial Intelligence [AI] Act [59]), and security frameworks—including robust technical decisions, penetration testing, and International Organization for Standardization certifications—was a significant part of the development, ensuring that ROOM can be sustained beyond the research setting and avoid the “valley of death” phenomenon [60]. ROOM is fully hosted on the university’s cloud infrastructure, and student authentication protocols were tested and integrated into the app, allowing EUR students secure and free access to ROOM.

### Communication and Marketing Strategy for ROOM’s User Adoption

Resources were allocated for implementation, and an implementation officer and a communication officer were hired to coordinate the rollout of ROOM and its communication campaign at EUR. User research indicated the need for diverse outreach strategies: some students engage with well-being proactively, while others seek support primarily during challenging periods. Furthermore, regarding messaging and slogans, some students respond positively to personal development themes (eg, “Be the best you”), while others prefer more compassionate messaging. Student associations, sports clubs, and fraternities may be more receptive to messages incorporating humor and references to popular internet culture. Peer-to-peer recommendations are also perceived as highly relevant by students (see Table 3). Early adopters of ROOM play a key role in spreading information and creating a bottom-up movement to engage other students. Those less interested in well-being and personal development are likely to engage later, once ROOM gains popularity.

Therefore, ROOM’s implementation and communication strategy includes promotion through multiple channels, such as social media, the university intranet, the Student Wellbeing Programme’s communication channels, student ambassadors, and interactive installations displaying ROOM’s signature objects (eg, the Lava lamp) alongside QR codes directing students to the ROOM App Store page for more information and downloads. These initiatives are being rolled out through targeted campaigns across the academic year, ensuring that students are reached when they are most receptive—whether seeking support (eg, during periods of stress) or focusing on self-development (see Table 2). This approach is designed to meet students where they are, aligning with their diverse needs and readiness to engage with the tool.

### Ethical Consideration

All research activities conducted across the different phases of the road map adhered to the Netherlands Code of Conduct for Research Integrity and received approval from the Ethics Review Board of the Department of Psychology, Education, and Child Studies at EUR (records number 20-066a2, ETH2122-0677, and TH2324-0086). The final randomized controlled trial evaluating ROOM’s effectiveness was registered at ClinicalTrials.gov (NCT05576883). All participants in our studies provided informed consent after reviewing information about the specific round of data collection they joined. Privacy measures included data anonymization and secure storage. Participants were compensated with vouchers of varying amounts, generally reflecting the time invested in each study (eg, €5 [US $6.7] for 30 minutes). For smaller studies, participants were entered into a lottery as a form of compensation.

## Discussion

### Principal Findings

The objective of this paper was 2-fold: to present the developed solution, ROOM, and to share insights from its development process. Specifically, we detailed how the 4 phases of the CeHRes road map [39,40]—contextual inquiry, value specification, design, and operationalization—shaped this mobile intervention. The design process was iterative, employing a range of qualitative and quantitative research methods and engaging key stakeholders, including university students, mental health experts, study advisers, and university psychologists, throughout the development phases. The outcome of this process is ROOM, which incorporates the following signature features: it adopts a transdiagnostic approach to mental health, addressing the underlying mechanisms of a range of mental health issues common among university students by targeting ER and self-awareness. The intervention functions as a micro-intervention, promoting active engagement with its content both inside and outside the app. It also features a recommender system that adapts content to the user based on their preferences (eg, managing unhelpful thoughts or practicing relaxation) and momentary needs (ie, current emotional states). ROOM adheres to high standards of privacy and security, ensuring transparency in data management. It also gives users autonomy over their engagement with the app and deliberately avoids engagement patterns commonly seen on social media, promoting genuine and meaningful user interaction.

In the following sections, we discuss the resulting intervention, outline the development process, highlight the challenges encountered during the app’s design, and provide practical recommendations for developers creating adaptive mental health interventions for youth.

### The Developed Solution

ROOM employs a transdiagnostic approach to mental health, providing flexible, personalized support tailored to each student’s unique needs. This approach has gained popularity for its potential to prevent common mental health issues by targeting underlying vulnerability factors rather than focusing on disorder-specific symptoms [61]. Previous studies have shown that applying a transdiagnostic approach in digital interventions can successfully reduce anxiety and depressive symptoms—2 of the most common concerns among university students [62]. In ROOM, the transdiagnostic factor targeted is ER [63]. The use of adaptive ER strategies—such as recognizing, expressing, and appropriately regulating emotions—is associated with enhanced well-being [64] and improved academic performance [65]. Our studies indicate that engaging with ROOM reduces distress and enhances ER skills [55]. Focusing on ER among university students is a promising approach for maintaining and promoting their well-being.

Another key target of the ROOM intervention, closely linked to ER, is self-awareness. Awareness of one’s emotional state is essential for selecting appropriate ER strategies [66]. ROOM fosters self-awareness by encouraging users to monitor their emotional states and gain insights about themselves through questionnaires assessing various psychological states (eg, distress symptoms) and traits (eg, perfectionism) that influence well-being. Because of the psychoeducational content embedded in the self-assessment modules (as well as in the exercises), we expect ROOM to enhance users’ mental health literacy. Mental health literacy plays a key role in fostering help-seeking behaviors, encouraging users to recommend help to others, and reducing mental health stigma [67]. Thus, ROOM may help decrease stigma and promote help-seeking among students who require additional support. While this was not the focus of the current studies, it will be evaluated in future research.

This target population has a strong preference for systems that offer personalization [35,36] and aims to reduce screen time. Therefore, rather than protocolized, longer, and intensive mental health interventions such as internet-based CBT [68] or mindfulness-based stress reduction [69], ROOM incorporates brief (3-10 minutes) behavior change exercises that can be conceptualized as micro-interventions—that is, targeted, short interventions integrated into a person’s daily routine, causing minimal disruption [70]. These exercises provide practical, real-time strategies that help students regulate their emotions and enhance self-awareness in the moment. Research on micro-interventions suggests that this type of approach is a promising way to improve moods [71,72], promote value-based behaviors [73], and encourage behavioral activation [74]. Consistent with this, our studies indicate that engaging with ROOM has successfully improved momentary moods [55], fostered growth in ER skills, and reduced stress levels. However, the long-term effects of this intervention remain to be evaluated.

ROOM also incorporates an intelligent recommender system that delivers meaningful, context-sensitive suggestions based on users’ history, preferences, and current emotional states, ensuring relevant support in the moment. Consequently, ROOM users do not follow a predetermined regimen; their engagement with the content is personal and varies for each student, enabling genuine personalization. Recommender systems are playing an increasingly important role in mental health, serving multiple functions such as reducing choice overload, strengthening the digital therapeutic alliance (ie, the relationship between the user and the digital tool [75]), and enhancing user engagement [76].

A distinctive feature of ROOM is its engagement strategy. The app is not designed for long-term retention but aims to teach users relevant ER strategies and facilitate the application of these skills in real-life settings [43]. The app places less emphasis on traditional engagement metrics—such as time spent online (ie, micro-engagement)—and instead prioritizes meaningful interactions. It scaffolds engagement with the content and supports the associated behavior change process beyond the screen (ie, macro-engagement [45,46]). This design choice is grounded in our research, which indicates that digital screens often cause stress for students, prompting them to minimize screen time. This finding aligns with recent studies showing that users are increasingly saturated with online tools and experience digital stress [77,78]. Moreover, applying learned skills is an aspect that Generation Z considers crucial in their learning process [3,79]. ROOM incorporates learning transfer elements—such as implementation intentions [80], associative cues, and tactile guidance [81]—to support real-world application of skills, aligning with the values of this generation. Our studies have shown that the transfer elements may accelerate the practice of learned techniques in users’ daily lives [43], suggesting that engagement modalities extending interaction with the content beyond the app and into real-life activities represent a promising avenue for enhancing learning and behavior change outcomes.

Long-term, sustained usage is not a goal of ROOM. However, users need to engage with the app for a defined period for it to achieve its intended impact. To support effective engagement, ROOM incorporates persuasive design elements, including gamification mechanics and a recommender system—both recognized as key drivers of user engagement [82]. One gamification element in ROOM is the collectibles mechanic [83], in which users collect objects linked to exercises and use them to decorate and personalize their virtual room. This feature provides opportunities for self-expression (ie, personalizing their room [84]) and highlighting exercises they enjoyed, while also offering quick access to revisit those exercises, addressing users’ need for time efficiency. Collectible game mechanics are a popular gamification element [83], and formative evaluations indicated that users greatly enjoyed this feature. The second gamification element is the 21-day challenge, which encourages users to engage with the app consistently for 3 weeks to experience its benefits. Although this element has not yet been formally assessed, previous research suggests that challenges can effectively motivate users to continue engaging with a system or application [85].

Lastly, user autonomy and privacy emerged as central themes in our research, consistent with prior studies on this population [35,37]. ROOM supports autonomy by allowing users to engage flexibly at their preferred pace and in their chosen manner, while also incorporating features that facilitate learning transfer [43] and maximize positive outcomes. The app is fully transparent about how user data are managed and provides clear insights into the workings of its recommender system. Many commercial apps lack robust privacy standards and may sell users’ mental health data to third parties [86]. By contrast, ROOM stands out by effectively balancing persuasive strategies with user autonomy while upholding strict data privacy standards.

### The Development Process

ROOM is the result of a 4-year development process guided by the CeHRes road map [40]. Throughout this period, the target population and other relevant stakeholders were continuously involved to ensure the app aligned with users’ needs and preferences. We employed a range of research approaches, from UX studies to experimental research, to design and refine the app’s content and functionalities. Using both quantitative and qualitative methods deepened our understanding of the student population, their needs and preferences, how they engage with interventions independently (ie, in their private time), and their perceptions of these interventions. By identifying problems early and iteratively optimizing the design, ROOM’s content and functionality became better aligned with the target users, enhancing both its UX and effectiveness. Moreover, collaboration with experts from diverse disciplines—each contributing unique perspectives, expertise, and methodologies—strengthened problem-solving, fostered innovation, and supported the development of comprehensive solutions throughout the project [87]. Their involvement was crucial for evaluating content for fidelity to the intervention approaches, addressing issues identified in user research, and designing user-friendly, engaging app elements that support behavior change within the available resources.

### Strengths and Limitations

Currently, evaluation of the intervention’s effectiveness and uptake is ongoing [42], and ROOM is being implemented university-wide, achieving over 3200 downloads within the first few weeks. While results from the final effectiveness study [42] are not yet available, our research already provides preliminary support for our approach to digital intervention development. Particularly, ROOM is generally well received by students, functions as intended, and is safe to use, as indicated by improved or stable levels of distress symptoms [55]. The main strength of our approach lies in its iterative, agile development, the inclusion of a variety of stakeholders, and, most importantly, the consistent consideration of information gathered from our target population (ie, university students).

A notable limitation of our development process is the overrepresentation of female participants in these studies. This is unsurprising, as female students often report experiencing more mental health difficulties [4]. Moving forward, we plan to actively involve more male and other gender participants in the development process (eg, when adding content or modifying the visual design) to enhance the inclusivity of ROOM and ensure it incorporates perspectives and information that may currently be underrepresented.

### Challenges, Compromises, and Recommendations

Developing ROOM required navigating multiple challenges, including balancing student needs with constraints such as privacy concerns and interdisciplinary collaboration, all of which influenced the app’s final design and functionality. Below, we outline key challenges and compromises made, along with practical recommendations.

#### Depth of Information Versus Preference for Brevity

Adapting mental health interventions for Generation Z, who prefer short and engaging content, led us to develop brief 3-10-minute behavior change exercises. The challenge was maintaining the depth and quality of psychological content within this condensed format. Through iterative testing and expert input, we achieved a balance between brevity and preserving essential content. For interventions of this type, it is recommended to use a streamlined flow that presents only the necessary information to complete the exercise, while offering optional “learn more” buttons for users who want additional details or support. Developers should continuously test content with users and consult experts (ie, mental health professionals) to ensure fidelity to therapeutic principles and maintain exercise quality.

#### Content Diversity Versus Interactivity

Balancing content diversity with interactivity posed another significant challenge. While our goal was to offer a wide range of content to meet the varied needs of the target population, prioritizing content variety within the iterative development process limited our ability to incorporate highly interactive elements, such as videos or interactive gameplay, due to resource constraints. Currently, the app primarily relies on written content, quizzes, and audio guides. Given that this target population often learns through visual media (eg, videos) [3] and is accustomed to high levels of interactivity in digital tools, incorporating varied media formats—especially videos—is strongly recommended when developing digital solutions for youth. If such formats are not feasible, presenting content in brief segments, such as 2 sentences per page, can serve as an effective alternative.

#### Privacy by Design Versus System Interactivity and Personalization

Designing for privacy while maintaining system interactivity and personalization posed another significant challenge. Unlike many commercial apps that prioritize UX over privacy [88], ROOM adhered to the “privacy-by-design” principle, reflecting both the sensitivity of mental health data and Generation Z’s strong privacy concerns [35]. We opted to store and process user data locally on their phones rather than in the cloud, ensuring compliance with privacy regulations such as the EU GDPR law [58]. This approach, however, limited the level of personalization that could otherwise be offered. For example, we forwent passive data collection, such as GPS tracking, which could have enabled more tailored recommendations and the design of just-in-time adaptive interventions [89]. However, the use of federated machine learning [90] enabled us to provide a personalized experience while complying with strict privacy-by-design principles. It remains essential to continue protecting user privacy and promoting ethical practices by exploring innovative, privacy-preserving technologies. Future research should investigate the balance between personalization and privacy, examining user attitudes and the effects of these trade-offs.

#### Fixed Resources Versus Iterative Process

Another challenge was operating within fixed time, budget, and scope constraints while committing to an iterative development process, in our case following the CeHRes road map, and using agile project management [91]. Agile project management offers various strengths, such as an iterative and flexible approach to product development, but it also presents challenges, including limited process visibility, difficulties with long-term planning, and resource allocation issues [92]. In our development process, we struggled with resource allocation and had to continuously adjust the project scope [91]. Balancing technical requirements for a sustainable product, UX, ethical guidelines, and scientific rigor made the prioritization of values and features paramount. We implemented regular review checkpoints to assess progress and remained adaptable to evolving requirements within the existing resource constraints. We recommend that developers carefully select an appropriate project management approach and conduct regular budget reviews after each iteration (eg, monthly). These reviews should include refining priorities and ensuring that remaining resources are focused on high-priority items, helping to keep development on track within budget limits.

#### Interdisciplinary Teams and Collaboration

Working with interdisciplinary teams and across sectors (ie, academia and industry) required developing a shared language and vision—both a challenge and an opportunity [93]. While the diverse expertise brought by team members from different fields enriched the project, it required time and effort to synchronize our understanding and approaches. For example, academic-industry collaborations are crucial for building viable products that can survive “in the real world,” but they face challenges due to differing processes and pace [94,95]. Academia operates under rigorous, protocol-driven environments that prioritize deliberative, gradual progress, often with changing requirements, whereas industry settings demand speed, innovation, and rapid implementation [95]. To bridge these differences, it is paramount to allocate time at the start of the project to select an appropriate collaboration model, align processes, and ensure that neither the integrity nor the innovativeness of the project is compromised.

### Conclusions

This paper presents the multifaceted process behind the development of a mobile transdiagnostic well-being intervention for university students. Several key insights emerged that may inform the development of future digital mental health tools for young people. First, our findings suggest that for a heterogeneous population such as university students, a transdiagnostic approach to mental health can effectively address their varied needs. Second, tech-savvy Generation Z often seeks to reduce screen time due to technostress, highlighting the importance of designing digital mental health tools that support both in-app and off-screen engagement strategies. Third, system responsiveness and content curation are highly important to users, highlighting the need for adaptive interventions and the integration of tools such as AI-based recommender systems to effectively meet their needs. Fourth, prioritizing security and privacy in digital mental health tools is essential for building and maintaining users’ trust. This project demonstrates that, even when creating a “data-hungry” system, privacy-friendly practices are achievable when integrated from the outset. Lastly, developing such a tool requires user-centered approaches and a strong interdisciplinary team to address the design, functionality, and content needs of stakeholders. Although the full evaluation of ROOM’s impact is still pending, initial feedback indicates that it is well-received, feasible, and safe for student use.

## Appendix

Multimedia Appendix 1 Overview of Centre for eHealth Research (CeHRes) stages, its objectives, and related activities.

## Abbreviations

ACT: acceptance and commitment therapy
AI: artificial intelligence
CBT: cognitive behavioral therapy
CeHRes: Centre for eHealth Research
DBCI: digital behavior change intervention
ER: emotion regulation
EU: European Union
EUR: Erasmus University Rotterdam
GDPR: General Data Protection Regulation
OSF: Open Science Framework
UX: user experience

## Data Availability

The data supporting the findings of the design process will be made available on the OSF [43] following an embargo period after publication, to allow for the commercialization of the research findings. Data from qualitative interviews, however, will not be shared to protect participants’ privacy and confidentiality.
