# Supplementary material for: ROOM to Grow, a Mobile Well-Being Intervention for University Students: Overview of the Design Process and Outcomes
Source: JMIR Form Res. 2025 Dec 17;9:e63325. doi: 10.2196/63325 (PMC12756657; doi:10.2196/63325)
Supplement: Multimedia Appendix 1 [file formative_v9i1e63325_app1.docx]

# Appendix 1. Overview of CeHRes stages, its objectives, and related activities

Table 1 presents information on 5 development phases for e-health tools derived from the Centre for e-Health Research (CeHRes) Roadmap [(Hanneke et al., 2024)]

Table 1. Overview of CeHRes stages, its objectives, and related activities

| Stage | Objective | Activities |
| --- | --- | --- |
| Contextual Inquiry | Understand the problem, including target behaviour or health outcome and existing solutions.  Understand the context. Identify barriers and opportunities of the ecosystem for a digital tool. How do the target users relate to and behave in the context?  Identify the stakeholders and qualify their involvement. Who has an influence on or is affected by the problem and plan when and how to involve them? | The process starts with thorough research to understand the problem space, including but not limited to:   - Desk research - Focus groups/interviews with key stakeholders - Observation/diary studies - Stakeholder mapping |
| Value Specification | Creating guidelines to direct the process in a way that the needs of the stakeholders are addressed throughout the process. | Specifying what would be valuable for each stakeholder. These values, alongside the needs and wishes of the target users, are then translated into different types of requirements for the digital intervention and surrounding (eco)system. |
| Design | Using the requirements as a guide, create a series of mockups/prototypes to validate and perfect design concepts, ideas, and interactions. Rapid prototyping and testing are done with different, increasingly more complex versions.  Test the relevance, acceptability and appropriateness, preliminary user experience, and usability/ease of use (when possible) of the DBCI concept with all the stakeholders | Depending on the stage of the design, researchers can conduct a series of protocols to shape and validate the solution. Including but not limited to:   - Thinkalouds - Co-design sessions - Cognitive walkthroughs - Usability testing - Diary studies - A/B tests |
| Operationalization | Production of the final, complete prototype. Understand the ecosystem of the digital intervention and implement the technology in the context effectively. | At this stage, research is carried out to align stakeholder expectations, and the comprehensive implementation of the digital tool is carried out. |
| Summative Evaluation | eHealth technology's usage and its impact on the target users and the system where the technology is embedded, are evaluated | To evaluate the solution, evaluative research is carried out. Including but not limited to:   - Interviews - Randomized controlled trials - Ecological momentary assessments - N=1 studies - Continuous performance monitoring (e.g, usage and system logs) |
